# Supplementary material for: Chromothripsis during telomere crisis is independent of NHEJ, and consistent with a replicative origin
Source: Genome Res. 2019 May;29(5):737–49. doi: 10.1101/gr.240705.118 (PMC6499312; doi:10.1101/gr.240705.118)
Supplement: Supplemental Material [file supp_gr.240705.118_Supplemental_file_1.zip › contigs/annotated_contigs/DB79/contig.2.DB79_length_423_mean_cov_4.25531914894.docx]

**DB79_length_423_mean_cov_4.25531914894**

```
TTCAGTTCCCAGTGATCTTACATTAAACATTTGTCTGTCTAAAACAATAGGTTAACTATAGCCAAATTATAACCATACCATTCCTCTCC
 >chr7:116338463-116338697 + E=2e-129 p=2e-02
ACACAAAATCCTATAAACAGCATGTGATCATATTGCTTCTAGAATTTATGATTGTTTTCTTCCAAAAGGAAGCTAAATTTAGCCTAGTA

ATTCTACATGCGCTCAAGAAAACAATGCCTGCTGTGATTTCTAGAATAAATGAAT|G|ACAAAGATGGTCTCTTTATTTACTTGAATAA
                                                        >chr7:116331241-116331431 - E=7
TTACTTTAAATATTTCACTTTGCATTTGAAAACATATCTATTAGTTAAGATTTCTGAGGTGGTATACTAGTATTTTCTTATTATATAAT
e-100
TCTAAAATCTTCTACAATATTTACATAAGATAATCTCAAAATGGAAATAATGGATCTAAGTAAAGAGTT
```
